# The impact of oxytocin administration on brain activity: a systematic review and meta-analysis protocol

**DOI:** 10.1186/s13643-016-0386-2

**Published:** 2016-11-29

**Authors:** Daniel S. Quintana, Tim Outhred, Lars T. Westlye, Gin S. Malhi, Ole A. Andreassen

**Affiliations:** 1 NORMENT, KG Jebsen Centre for Psychosis Research, Division of Mental Health and Addiction, University of Oslo and Oslo University Hospital, Ullevål, Kirkeveien 166, PO Box 4956 Nydalen, 0424 Oslo, Norway; 2 Academic Department of Psychiatry, Northern Sydney Local Health District, Sydney, Australia; 3 Sydney Medical School Northern, The University of Sydney, Sydney, Australia; 4 CADE Clinic, Royal North Shore Hospital, Northern Sydney Local Health District, Sydney, Australia; 5 Department of Psychology, University of Oslo, Oslo, Norway

**Keywords:** Oxytocin, Brain imaging, Systematic review, Meta-analysis, Protocol

## Abstract

**Background:**

Converging evidence demonstrates the important role of the neuropeptide hormone oxytocin (OT) in human behaviour and cognition. Intranasal OT administration has been shown to improve several aspects of social communication, such as the theory of mind performance and gaze to the eye region, and reduce anxiety and related negative cognitive appraisals. While this early research has demonstrated the potential for intranasal OT to treat psychiatric illnesses characterized by social impairments, the neurobiological mechanisms are not well known. Researchers have used functional magnetic resonance imaging (fMRI) to examine the neural correlates of OT response; however, results have been variable and moderating factors are poorly understood. The aim of this meta-analysis is to synthesize data examining the impact of intranasal OT administration on neural activity.

**Methods/design:**

Studies that report fMRI data after intranasal OT administration will be identified. PubMed, Embase, PsycINFO, and Google Scholar databases will be searched as well as the citation lists of retrieved articles. Eligible articles written in English from 2005 onwards will be included in the meta-analysis, and corresponding authors of these papers will be invited to contribute *t*-maps. Data will be collected from eligible studies for synthesis using Seed-based *d* Mapping (SDM) or Multi-Level Kernel Density Analysis (MKDA), depending on the number of usable *t*-maps received. Additionally, publication bias and risk of bias will be assessed.

**Discussion:**

This systematic review and meta-analysis will be the first pre-registered synthesis of data to identify the neural correlates of OT nasal spray response. The identification of brain regions underlying OT’s observed effects will help guide future research and better identify treatment targets.

**Systematic review registration:**

PROSPERO CRD42016038781

**Electronic supplementary material:**

The online version of this article (doi:10.1186/s13643-016-0386-2) contains supplementary material, which is available to authorized users.

## Background

The neuropeptide oxytocin (OT) has attracted significant scientific and lay interest for its role in social cognition and behaviour [1, 2]. For example, a single administration of OT has been shown to modulate the perception of social cues [3], motivate in-group cooperation [4], increase gaze to the eye region of faces [5], and reduce anxiety [6, 7]. Due to these reported cognitive and behavioural effects, researchers have begun investigating OT’s potential to treat psychiatric conditions, such as autism spectrum disorders, schizophrenia, and social anxiety disorder in a number of clinical trials (for a review, see [8]).

Although the modulatory effects of OT on social behaviour and cognition have been demonstrated repeatedly [8, 9], the mechanisms are poorly understood [10, 11]. To better elucidate these behavioural and cognitive effects, researchers have investigated the neural correlates of OT’s effects using brain-imaging tools such as functional magnetic resonance imaging (fMRI). Converging evidence from this field suggests the amygdala—a key brain region involved in the processing of emotional [12] and social stimuli [13]—is an important target of OT administration [14–18]. Although the amygdala has received significant research interest, other areas of the brain have also implicated in OT’s response [19].

**Fig. 1:**
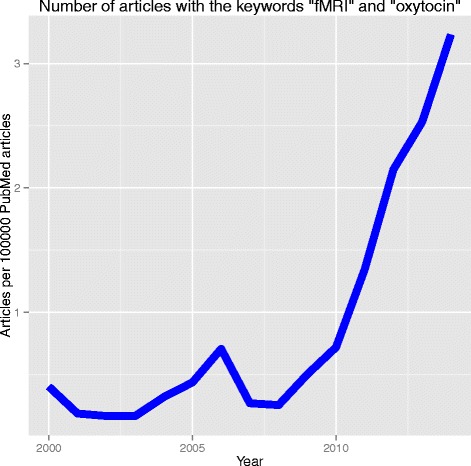
Increasing interest in fMRI and oxytocin. PubMed metadata was collected on the number of articles containing the terms “fMRI” and “oxytocin” published between 2004 and 2014. A loess smoothed fit curve was overlaid on data points to illustrate the year-to-year trend. Data was collected using the “RISmed” R package

Interest in the use of fMRI to understand the effects of OT has been increasing exponentially in the past decade (Fig. 1), with a total of 115 publications using the keywords “oxytocin” and “fMRI” published between 2004 and 2014 (although only a minority of these publications specifically assess the impact of OT administration on fMRI outcomes). Early work primarily investigated neural activity during emotional task processing after OT administration [14, 16, 20]; however, recent research has begun to investigate resting state activity and connectivity [21, 22]. Research is yet to synthesize studies that explore resting state neural activity, which is important for understanding neural modulation with OT regardless of task, particularly within the context of heterogeneity in task design. Meta-analysis provides a robust statistical method of synthesizing effect sizes across studies and is a valuable tool for clarifying past findings.

Various methods are available for the meta-analysis of brain-imaging data [23]. Prior research synthesizing OT fMRI data has adopted a meta-analytic approach using effect size signed differential mapping [24]. This approach uses extracted fMRI peak coordinates to create an *estimated* map of all possible brain region coordinates, which are then synthesized. However, this approach can bias effects of interest estimates where individual studies have reported a relatively high number of peaks when compared to others by chance. Additionally, it is difficult to analyse and interpret the impact of key study design characteristics such as dose and sex using this approach, unless a large number of studies are available. The impact of these problems on effect size-based meta-analysis results is difficult to determine within the existing limitations of the literature; therefore, meta-analysis methods that produce interpretable consistency measures are likely to provide guidance for future experimental investigation in this area. Given this is the ultimate goal, the Multi-Level Kernel Density Analysis (MKDA) coordinate-based approach is suitable [25] in that it provides clear interpretability and is unbiased by number of within-study peaks (see more details below). However, an updated version of effect size signed differential mapping—Seed-based *d* Mapping (SDM)—has been released [26], which can combine reported coordinates and *t*-maps, which are statistical parametric maps displaying the *t* statistic after estimation of the experimental parameters of interest for a given study. The *t* statistic is estimated at each voxel—the small volumetric unit of the statistical parametric map (e.g. 2 mm^3^)—for the whole brain, which in the context of performing a meta-analysis allows estimation of effect sizes at each voxel. By increasing the number of *t*-maps entered into the analysis, this approach has increasing sensitivity while reducing false positives [26]. The use of *t*-maps is preferable, as the inclusion of studies with reported coordinates requires the statistical estimation of unreported voxels. However, this approach relies on the availability of *t*-maps (e.g. as supplementary material or by direct researcher request). Therefore, if studies have a significant number of usable *t*-maps available (i.e. which will significantly increase sensitivity and decrease false positives), we will employ SDM in the first instance. Once data is extracted from eligible studies, maps of *d* values [27] and their variances are then created for meta-analysis (see below for further synthesis details).

## Methods/design

### Aims

The aim of the present study is to examine the effects of OT administration on human brain activity by synthesizing data from available research studies. This protocol is registered with PROSPERO (CRD42016038781) and has been reported here according to PRISMA-P [28] guidelines (see Additional file 1). Pre-registration of the analysis protocol will also help avoid potential bias by providing documentation of a priori analysis plans [29]. If protocol amendments are required, the PROSPERO registration will be updated.

### Inclusion and exclusion criteria

In this meta-analysis, we will include studies that meet the following criteria: (a) The study measured blood oxygenation level-dependent response using fMRI to assess response after OT administration; (b) the study provides standard Talairach or Montreal Neurological Institute (MNI) coordinates, allowing for comparison of findings; (c) the study includes a placebo comparison group; and (d) the study was written in English. A range of study designs (e.g. crossover, between-subjects) will be considered for inclusion as well as articles from the grey literature (e.g. pre-prints). Study authors will be contacted if any information germane to study inclusion is unclear.

### Search strategy

We will conduct a systematic literature search to collect studies that explore the neural effects of OT administration. Searches will be performed in PubMed, Embase, PsycINFO, and Google Scholar with the following combination of terms which were developed in consultation from two academic libraries: (“oxytocin” OR “syntocinon”) AND (“fMRI” OR “brain imaging” OR “functional magnetic resonance imaging” OR “MRI” OR “magnetic resonance imaging”). The search will be limited to articles published from 2005 (first fMRI OT study; 20) onwards. In a second iteration, reference lists within studies will be examined for remaining studies that include the critical measures.

### Data extraction and management

Two independent reviewers will independently scan primary titles to select articles for further scrutiny, deleting any duplicate titles. Abstracts of potentially eligible studies will then be read to determine eligibility for coding into a spreadsheet. When the title and abstract cannot be rejected, the full text of the article is obtained and reviewed for inclusion using a coding form. Any disagreements will be adjudicated by a third reviewer. If both reviewers agree that the trial does not meet eligibility criteria, it will be excluded. The two reviewers will then extract data from all eligible studies using a data extraction form. The coding forms will be developed specifically for this study, based on a pilot review, extraction, and calibration of five randomly included studies. Any disagreements regarding data extraction will be solved via discussion with a third reviewer. Data from studies initially selected based on title and abstract and articles included in the review will be documented. Reasons for the exclusion of retrieved articles will also be recorded for eventual documentation in a study search and data extraction flow diagram.

Available coordinates will be extracted from eligible papers and entered into a data collection form. This form will include (a) general information on studies including authors and titles; (b) information about the participants including, gender, age, and physical and mental health status; (c) information about the level the study on other moderator variables, including study type, experimental paradigm, and cognition modality (e.g. visual, auditory); and (d) information concerning study characteristics (e.g. publication year) and the risk of bias measures as defined by the Cochrane risk of bias tool. Corresponding authors from eligible studies will be contacted in order to request and obtain *t*-maps. In order to review and synthesize studies qualitatively, reported coordinates and peaks from *t*-maps (if available) will be plotted in MNI space for visualization and discussed in light of the methodologies employed and the authors’ conclusions.

### Risk of bias and strength of evidence

The Cochrane risk of bias tool will be used to assess risk of bias [30]. This tool encompasses six domains: selection bias, performance bias, detection bias, attrition bias, reporting bias, and other bias (i.e. bias problems not covered in the other domains). A table describing risk of bias across these domains for each included study will be provided to assess risk of bias within studies, as recommended [31]. The strength of evidence will be assessed and reported using the GRADE system [32].

### Statistical analysis

**Fig. 2:**
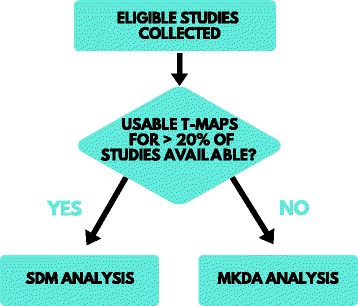
Decision-making process for choice of meta-analytic approach. *SDM* Seed-based *d* Mapping, *MKDA* Multi-Level Kernel Density Analysis

A decision-making process for choice of quantitative statistical analysis is outlined as follows (Fig. 2). Should *t*-maps be obtained, a combined coordinate and effect size-based meta-analysis using SDM will be possible; if not, a coordinate-only analysis using MKDA will be employed. In order to choose between approaches, a significant number of studies with available *t*-maps will need to be drawn, particularly given the likely low number of eligible studies available. Thus, if usable *t*-maps for 20% of studies are received and the analysis suits pooling data from the identified studies (given potentially differing methodological considerations), SDM analysis [26] will be performed in the first instance. If less than 20% of studies have associated *t*-maps, a coordinate-only analysis will be performed using MKDA, after consideration of examining the identified studies for suitability for pooling [33, 34].

SDM is an effect size-based approach that has been used to pool coordinate and *t*-map data largely from patient and control groups [35, 36]. If SDM is chosen, analysis will be performed using the SDM tool package (http://www.sdmproject.com). Available *t*-maps are simply converted to effect size *d* maps, and when only coordinates are available, an unnormalized Gaussian kernel (where a full width at half maximum is set, initially, 20 mm as recommended) is used to estimate effect size of voxels around the peak, which itself can is the only effect size that can be exactly calculated. Here, a random effects model would be implemented and carried out as recommended [26], as applicable to pooling the identified studies. Each study will be weighted by the inverse of the sum of its variance plus the between-study variance using the DerSimonian-Laird estimator [37], which gives greater weight to studies with smaller variance or larger sample size. The null hypothesis for SDM is that effect sizes are randomly distributed throughout the brain. Given that different *t*-maps will be thresholded with different correction methods, a combination of thresholds is recommended. Initially, an uncorrected threshold of *p* = .005 allows analysis of the robustness and heterogeneity of the findings with increasingly conservative thresholds.

MKDA is a coordinate-based approach that has been previously used to assess the impact of psychopharmacological agents on neural activity [38]. The MKDA statistic reflects the number of nominally independent contrast maps (i.e. statistical parametric maps from individual studies) that activate in the vicinity (e.g. within 10 mm) of each voxel in the brain; the null hypothesis is that the activation “blobs” from individual contrast maps are randomly distributed. Thus, a significant result indicates that more contrast maps activate near a specific voxel than expected by chance. If MKDA is chosen, analyses will be performed in MATLAB, using the MKDA tool package [25; http://wagerlab.colorado.edu/files/tools/meta-analysis.html]. MKDA difference analysis will be conducted to directly contrast the OT and task type conditions. The threshold for statistical significance will be determined using a Monte Carlo simulation (5000 iterations) and provided family-wise error rate correction for multiple comparisons at *α* < .05 corrected.

With the employment of either primary approach, moderators will also be entered in as covariates in meta-regression analyses. Where applicable, a Jackknife sensitivity analysis—where the same analysis will be repeated excluding one data point at a time—will determine if results are replicable. Given concerns surrounding publication bias in biobehavioural oxytocin research [39], a funnel plot of meta-analytic peaks will be constructed and analysed in order to determine publication bias in the collected sample as outlined by Egger and co-workers [40]. Moreover, between-study heterogeneity will also be assessed by constructing heterogeneity *Q*-maps (and corresponding *p* values). These maps will reveal brain regions that show significant between-study heterogeneity.

### Moderators

Many studies have explored the effects of OT on brain activity both during a task and at rest; however, the specific methods vary between studies. These methodological aspects include participant characteristics, experimental paradigm, OT dosage, and fMRI-related methodological differences. Moreover, the year of publication and overall study quality may also influence study effect sizes. Thus, the following potential moderator variables will be examined a priori in this meta-analysis to account for heterogeneity in the literature.

1. Participants. The effects of OT may vary between healthy and clinical populations. Thus, we will examine whether population type acts as a moderator for effect sizes. Furthermore, we will examine whether effect sizes are moderated by gender and age of participants.
2. Experimental paradigms. These paradigms can vary in studies that investigate the effect of OT on brain activity during tasks. For instance, the primary modality assessed can include visual stimuli, auditory stimuli, and executive function.
3. OT dosage. While 24IU is the typically administered OT dose, other dosages are occasionally administered. Considering the dose-dependent effects of OT [3], dosage will be included as a moderator where applicable.
4. fMRI methodology. The potential moderating effect of imaging package [41] and field strength will also be investigated.
5. Year of publication. Early, more preliminary studies may potentially exhibit different effect sizes due to improvements in study methodology or publication bias [42], so year of publication is included as an additional moderator to potentially assess bias.

## Discussion

There is growing interest in the neural correlates of intranasal OT administration in an effort to better understand its cognitive and behavioural effects. However, there is little consensus on the specific brain regions associated with intranasal OT administration and the impact of moderators such as gender and experimental paradigm. The present protocol describes the first systematic review and meta-analysis of fMRI studies that investigate the impact of intranasal OT. The inclusion of *t*-map data will provide greater precision than a coordinate-only analysis. The confidence in the body of evidence will also be assessed by measures of study quality and publication bias. Identification of specific brain regions underlying the effects of OT will assist future research and help identify treatment targets.

## Additional file

Additional file 1: PRISMA-P (Preferred Reporting Items for Systematic Review and Meta-Analysis Protocols) 2015 checklist. Recommended items to address in a systematic review protocol. (PDF 160 kb)

## Abbreviations

fMRI: Functional magnetic resonance imaging
MKDA: Multi-Level Kernel Density Analysis
MNI: Montreal Neurological Institute
OT: Oxytocin
SDM: Seed-based *d* Mapping
